# Porphyrin Supramolecular Arrays Formed by Weakly Interacting Meso-Functional Groups on Au(111)

**DOI:** 10.3390/molecules24183326

**Published:** 2019-09-12

**Authors:** Esteban Sánchez-Muñoz, José L. Gárate-Morales, Jacinto Sandoval-Lira, Julio M. Hernández-Pérez, Rocío Aguilar-Sánchez

**Affiliations:** Facultad de Ciencias Químicas, Benemérita Universidad Autónoma de Puebla, 22 Sur y Av San Claudio CU, Puebla 72570, Mexico

**Keywords:** porphyrin, self-assembly, non-covalent interactions, Au(111)

## Abstract

The formation of a binary porphyrinic self-assembled system between meso-tetrakis(4-carboxyphenyl) porphyrin (TCPP) and meso-tetrakis(4-dimethyl amino) porphyrin (TDAP) was easily designed through non-covalent interactions in solution and adsorbed on a gold substrate. It was found that non-covalent interactions and geometrical conformations between porphyrins allow their self-assembly into a well-defined arrangement, which was confirmed by UV-Vis spectroscopy, electrochemistry, atomic force microscopy and density functional theory (DFT) studies.

## 1. Introduction

Facile electron transfer and photon connectivity are crucial factors to increase efficiency in molecular devices and new photonic materials that mimic photosynthesis and other phenomena in nature. The high degree of order within bidimensional (2D) supramolecular nanostructures may provide a basis to relate structural characteristics to electron and energy transport and to photon capture. The self-aggregation of porphyrinic systems plays a key role to mediate light harvesting and energy transfer processes [1]. Molecular assembly is a crucial part of several electron transfer processes important either in nature or in technological applications. An excellent way to develop functional materials is the formation of self-assembled systems into 2D predefined architectures based on non-covalent interactions between specific building blocks. Self-assembly is based on suitable interactions between molecules. On the one hand, the interactions should be sufficiently weak to allow the chemical entities to explore the surface energy potential and, on the other hand, interactions must afford appropriate strength to stabilize the desired structure as a whole molecular entity. Under this context, hydrogen bonding, acid-base, short-range and electrostatic interactions are important to define the construction of ordered chemical structures in equilibrium. Then, it is crucial to address a number of fundamental and experimental questions regarding the control of the assembly process, including electrostatic intermolecular interactions.

Porphyrins are well-known heterocyclic building blocks with a highly π-conjugated system, and they have a wide range of applications. The meso position of the porphyrin is one of the most reactive centers towards both nucleophilic and electrophilic reactions, so the periphery can be decorated with several functional groups that can offer interesting motifs for non-covalent interactions. The investigation of porphyrins and their derivatives has witnessed great progress in solution, yet the fabrication and fundamental understanding of well-ordered porphyrin molecular assemblies on surfaces to form 2D crystals is still a challenge.

With respect to the solution phase, an advantage of having 2D well-defined supramolecular assemblies on a surface is that, with a suitable design strategy, it is possible to interlink interesting optoelectronic, light emission, magnetic, catalytic, and photosensitizing properties for practical applications [2,3,4].

**Scheme 1:**
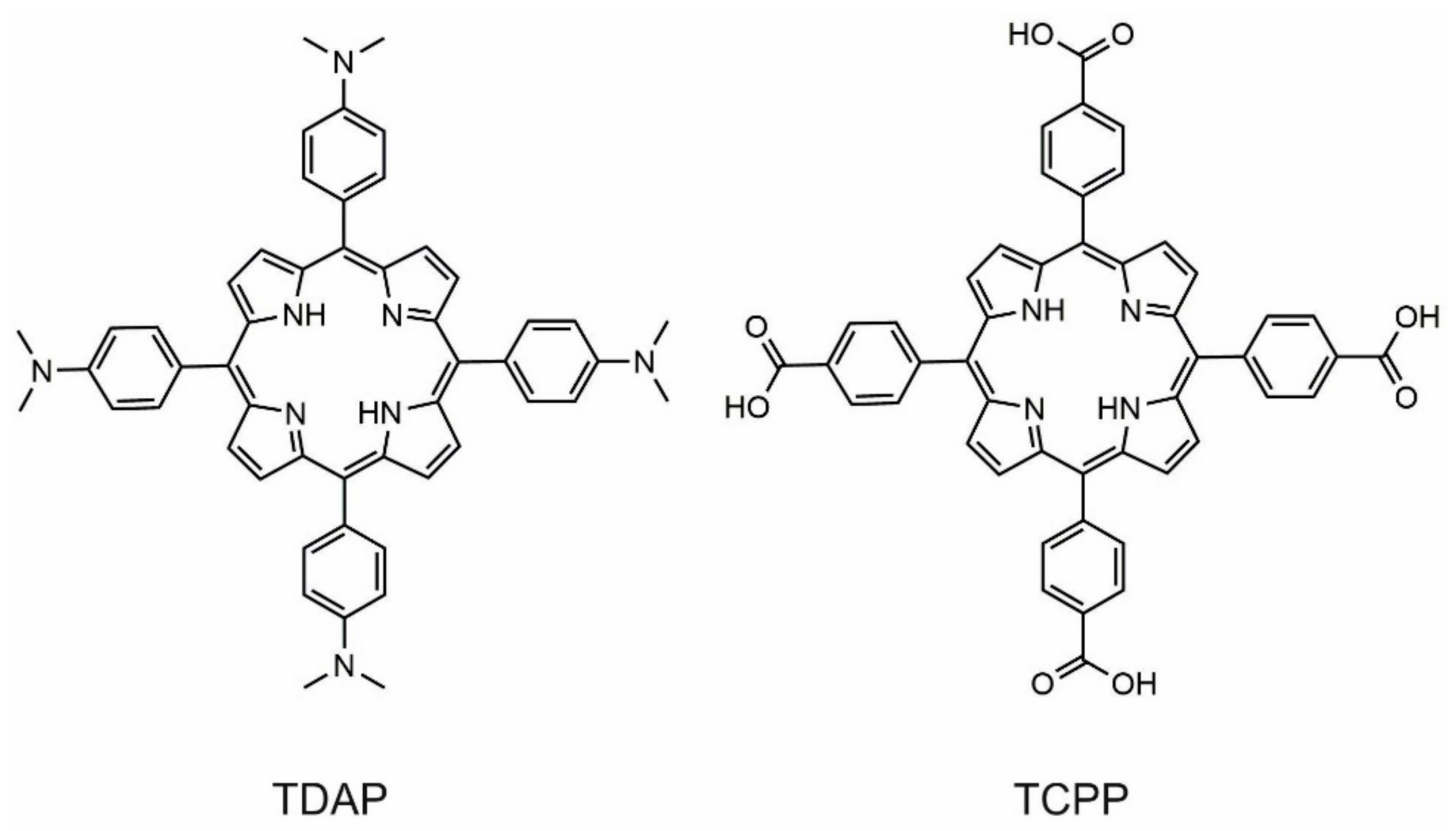
Molecular structure of meso-tetrakis(4-carboxyphenyl) porphyrin (TCPP) and meso-tetrakis(4-dimethyl amino) porphyrin (TDAP).

Since the direct synthesis of supramolecular assemblies implies complicated chemical routes [5,6,7,8], the non-covalent type of interactions looks like a more practical strategy to produce stable, well-ordered, self-assembled systems [9]. Porphyrins offer a unique opportunity to control the chemical architecture by combining hydrogen bonding [10], molecular recognition and electrostatic interactions [11]. In particular, meso-tetrakis(4-carboxyphenyl) porphyrin (TCPP) and meso-tetrakis(4-dimethyl amino) porphyrin (TDAP) (Scheme 1) are good building blocks for the formation of supramolecular compounds; their acid-base chemistry at the meso substituent permit the assembly of a chemical architecture by combining non-covalent interactions to allow a wide variety of structures due to a host-guest type system [12,13]. In one effort to design multiporphyrin arrays, we found that the assembly on solid surfaces is an essential first step for the rational design of molecular devices.

There have been some important studies [14,15] that report molecular assembly of porphyrins on solid surfaces under electrochemical control, and the importance of the surface potential on the molecular arrangement has been demonstrated. Nevertheless, in air, it has been difficult to find a report that reveals the existence of well-defined 2D porphyrin assemblies at the molecular level. 

In this work, we prepared assemblies of TCPP and TDAP in mild conditions by using an environment that allows the electrostatic interaction to occur in the meso positions of the TDAP and TCPP. UV-Vis, electrochemical techniques, atomic force microscopy (AFM) and density functional theory (DFT) studies demonstrated that the supramolecular assembly occurred between the meso groups of TDAP and TCPP through electrostatic interactions.

## 2. Results and Discussion

**Figure 1:**
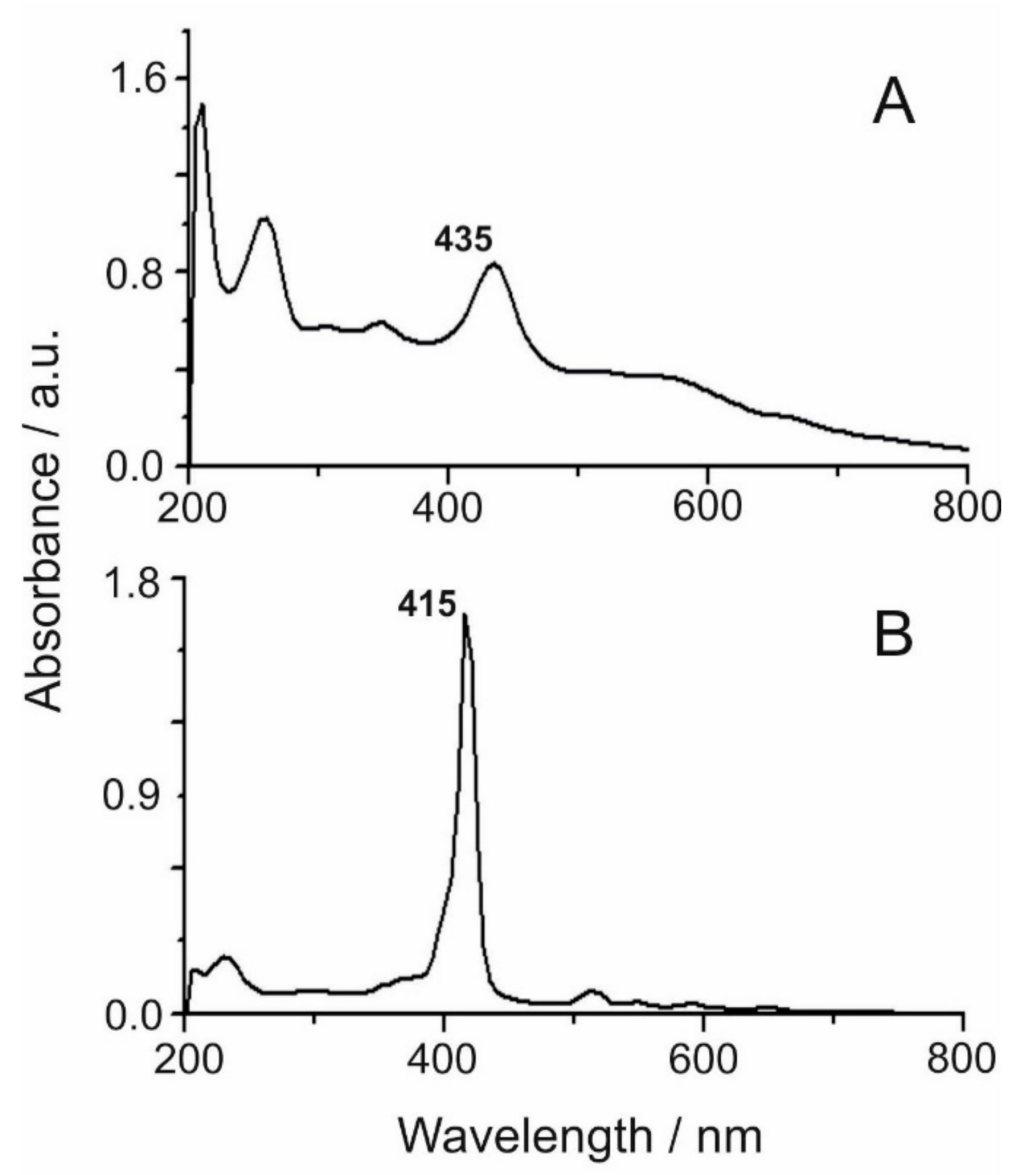
UV-Vis absorption spectra of (**A**) TDAP and (**B**) TCPP in ethanol.

The UV-Vis spectra of TDAP and TCPP porphyrins are shown in Figure 1. The strong transition to the second excited state of the free porphyrin (*Soret* bands) [16] is observed at 435 nm for TDAP (Figure 1A) and 415 nm for TCPP (Figure 1B). The Q-band region is notably well-defined at 520, 550, 595, and 645 nm for TCPP, while for TDAP, shoulder-like Q-bands are observed at lower energies, and the TDAP *Soret* band is slightly red shifted. The Q-bands in TCPP are indicative that the core of the porphyrin ring is unprotonated and is retaining the D_2h_ symmetry of the free base porphyrin [17,18]. It is important to mention that the carboxylic protons of the meso group are not interacting with the main core of the porphyrin. The differences in the UV-vis spectra of the two porphyrins reflect the dissimilarities in molecular symmetry and nature of the different substituents on the ring [16,19,20]. The extinction coefficients were ε = 21,642 cm^−1^ M^−1^ (TDAP, 435 nm) and ε = 136,664 cm^−1^ M^−1^ (TCPP, 415 nm), related to the good light absorption properties of the molecules.

**Figure 2:**
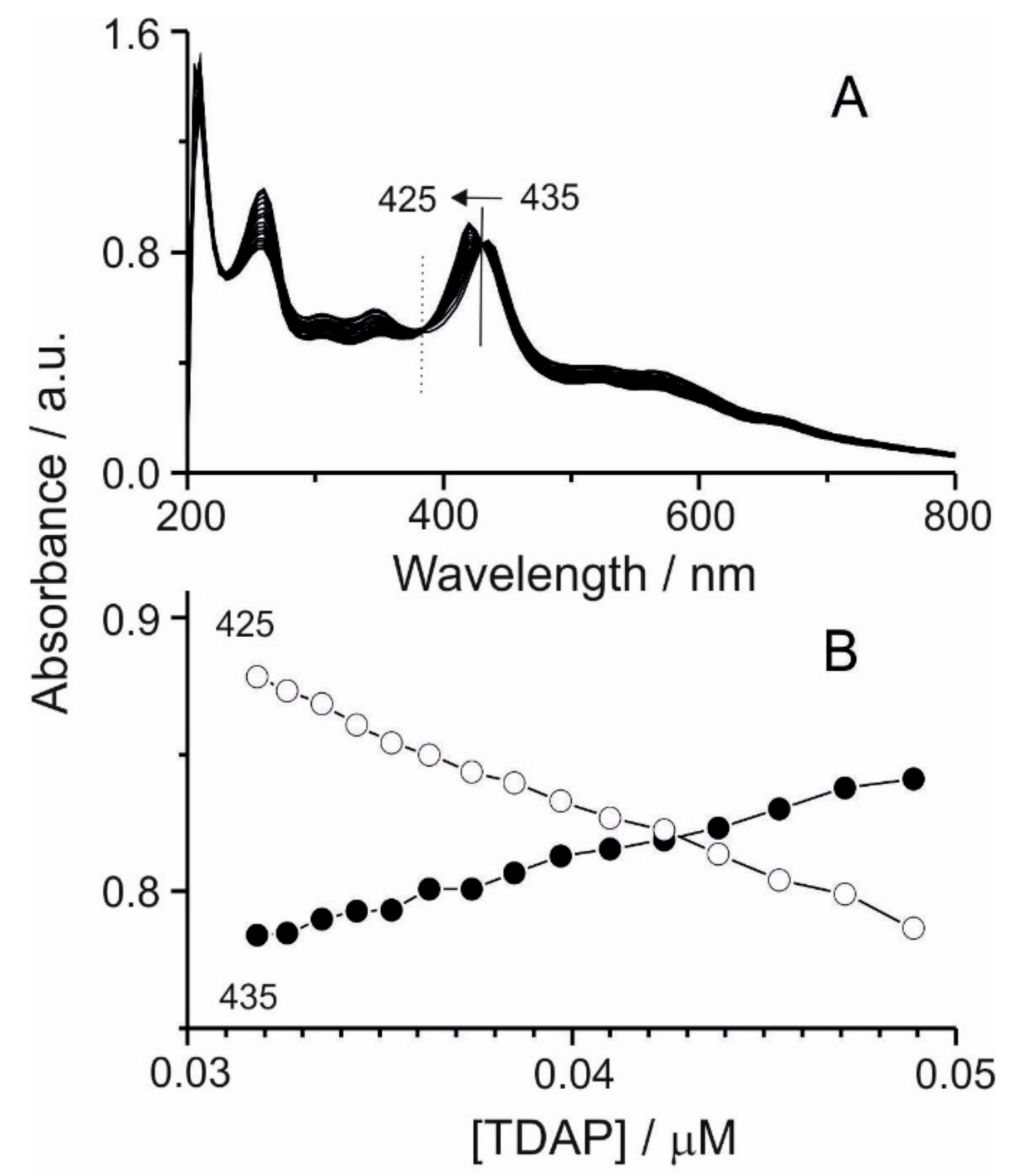
(**A**) Spectral changes obtained upon addition of TCPP to TDAP. Isosbestic points appear at 380 nm (dotted line) and 430 nm (solid line). (**B**) Evolution of absorbance at 425 nm (white dots) and 435 nm (black dots) as the TCPP concentration is increased.

Figure 2 shows the spectral changes and the corresponding titration curves of TDAP when TCPP was added. After the addition of TDAP, the *Soret* band presented a blue displacement from 435 nm towards shorter wavelengths, reaching 425 nm, and its intensity was increased. Blue shifting has been reported as a result of electrostatic interactions in the self-assembled nanoaggregates of TCPP in the presence of cationic surfactants containing a tertiary amine [21], and also due to the electrostatic interaction of porphyrins with polyelectrolytes as a result of the ionic assembly [22]. Since meso–meso covalently linked porphyrins showed individual *Soret* signals for each porphyrin in the UV-Vis [21] spectra, we consider that the blue shifting of the *Soret* band observed during the spectrophotometric titration of TDAP when TCPP was added is due to a weak electrostatic interaction between the two porphyrins.

By analysis of the isosbestic points [21], the stoichiometric ratio was determined to be 2:1 (TDAP:TCPP), providing additional evidence for the binding of the porphyrins. The titrimetric curve (Figure 2B) illustrates the strength and stoichiometry of the TDAP/TCPP interaction.

**Scheme 2:**
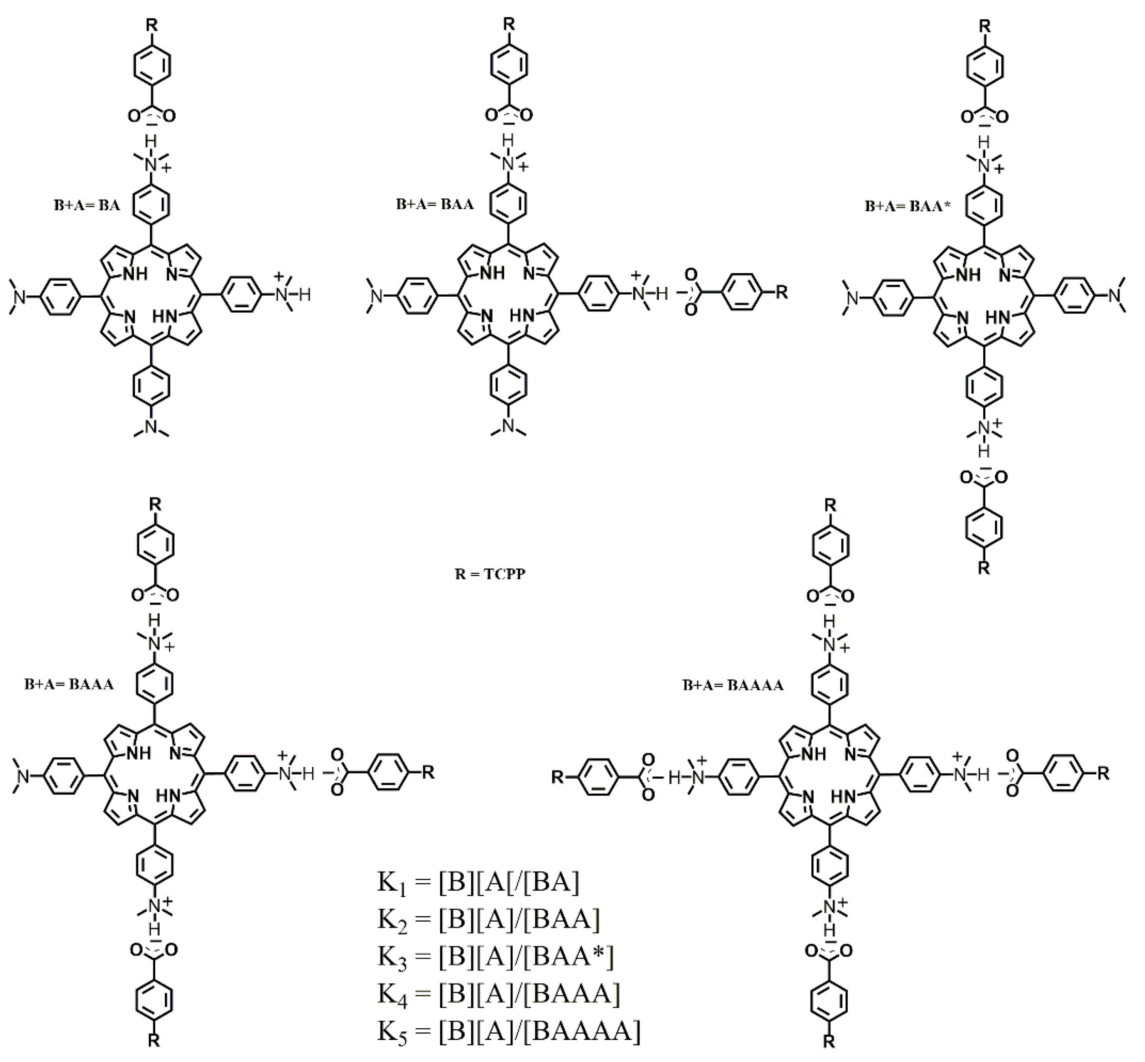
Proposed species generated during the addition of TCPP to TDAP.

The isosbestic points at 380 and 430 nm observed in the UV-Vis spectrum are associated with the equilibrium between the two porphyrins that form the molecular assembly, where the amino group of the TDAP and the carboxylic group of the TCPP in the meso position tend to form bonds by acid-base interactions in a polar solvent (i.e., ethanol) until an ionic form R-COO^−^-^+^NH(CH_3_)_2_-R is obtained. To explain blue shifting in our work, we propose that the base (TDAP) species can be readily protonated (B, BA (base-acid), BAA, BAA*, BAAA, BAAAA) when TCPP (acid) is added. Scheme 2 illustrates the proposed chemical species generated during the titration of TDAP with TCPP.

**Figure A1:**
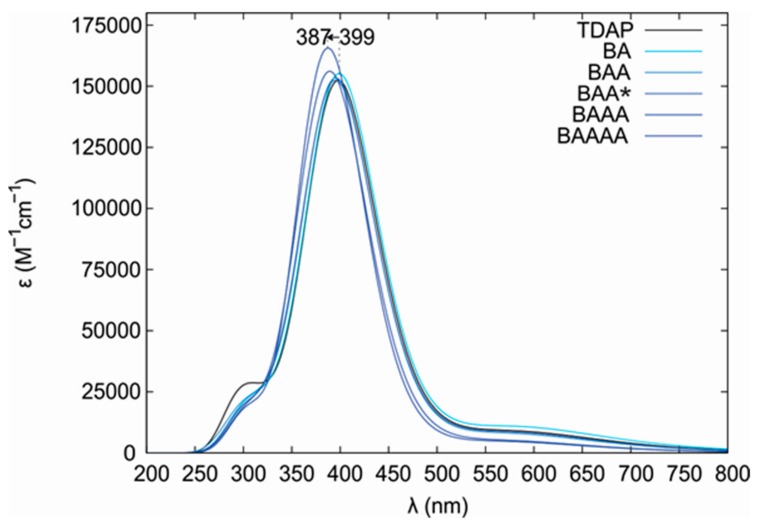
Theoretical spectra of TDAP titration with TCPP.

A computational analysis was carried out to investigate the interaction between TDAP and a proton (from carboxylic acid). Since the functional groups involved in the electrostatic interaction are a tertiary amine (-N(CH_3_)_2_) and a carboxylic acid (-COOH), for TDAP and TCPP respectively, the calculation was performed with a combination of TDAP and benzoic acid to facilitate the optimization. Figure A1 (Appendix A) represents calculated DFT absorption spectra for TDAP during titration with TCPP (represented by carboxylic acid), after the interaction with four acidic protons, i.e., when four molecules of benzoic acid are interacting with TDAP forming a supramolecular array.

The computational analysis predicts that the energy of the supramolecular TDAP-TCPP compound is higher (blue shifting) than the energy for the absorption of the TDAP alone, in accordance with the experimental results (Figure 2). It must be mentioned that in the DFT calculation, the contributions of the solvent and the intermolecular interactions were not taken into account. In order to estimate the strength of interaction between TDAP and TCPP, we calculated the interaction energy between these porphyrins in the gas phase. We found this interaction energy to be −14.95 kcal/mole (or −11.08 kcal/mole when using the counterpoise correction). The array TDAP-TCPP shows a hydrogen bond between N (TDAP) and O–H (TCPP) with a distance of 1.78 A. These characteristics suggest that this hydrogen bond is moderate [23].

**Figure 3:**
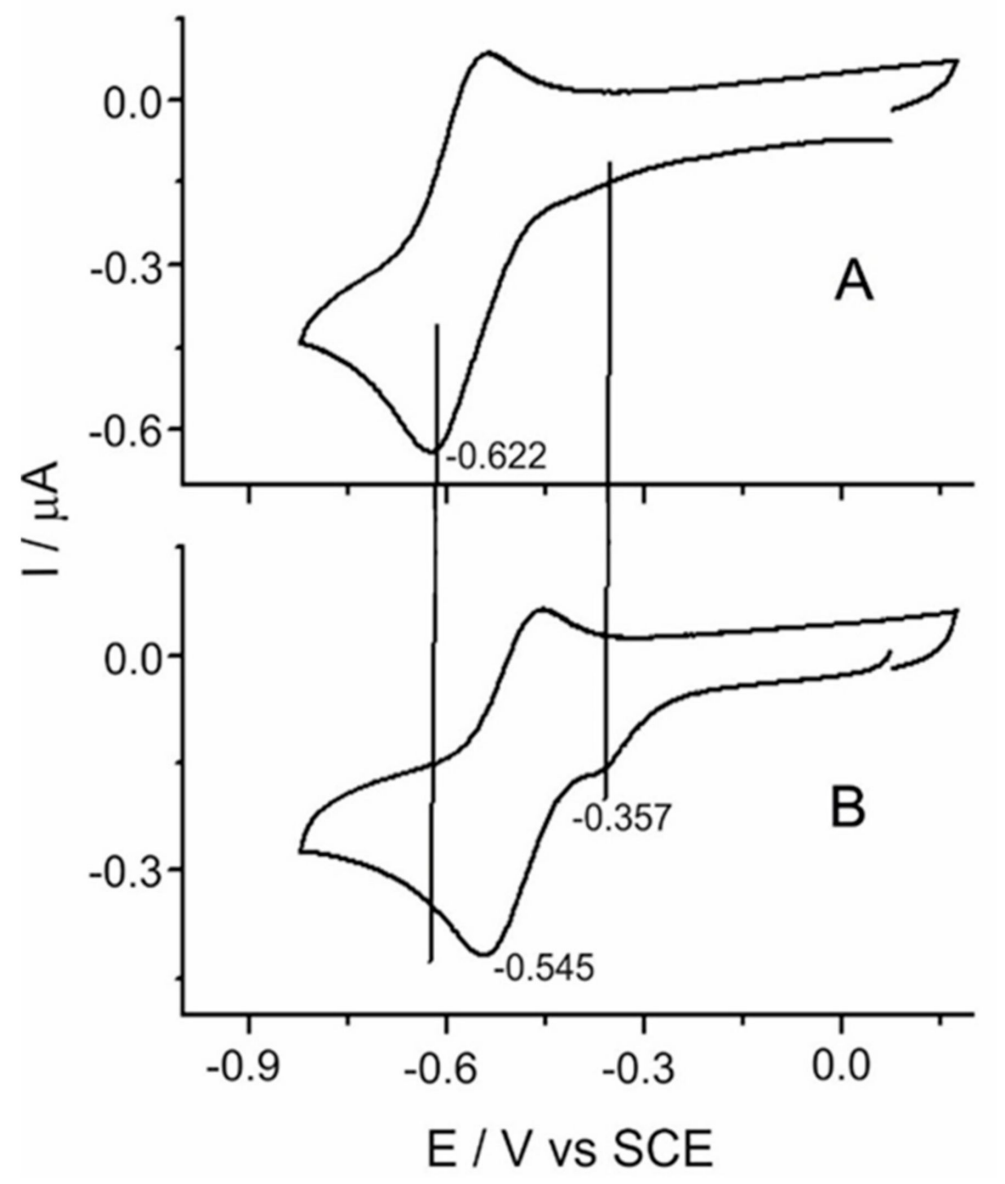
Cyclic voltammograms of (**A**) TDAP and (**B**) TDAP in the presence of TCPP, with both porphyrins at 1 × 10^−5^ M in DMSO and with 0.1 M TBAP as an electrolyte. Scan rate of 20 mV s^−1^.

The redox properties of TDAP and TCPP in non-aqueous media were investigated by cyclic and differential pulse voltammetry. Since the number of redox processes and the site of reduction and oxidation are highly dependent on the solution conditions, 0.1 mol/L tetrabutylammonium perchlorate (TBAP) in DMSO was chosen as a supporting electrolyte, given that under these conditions, the porphyrins tend to oxidize in serial steps of one electron [24], forming monocationic radicals and dication species. The same process occurs for the reduction, producing monoanionic radicals and dianions. Figure 3A illustrates the cyclic voltammogram for the reduction of TDAP. The experiment started at 0.1 V, and the potential was shifted to negative values. A well-defined redox reduction signal was observed at −0.622 V, which corresponds to the formation of the anion radical. Changing the electrode potential towards positive charge densities causes oxidation to occur at −0.531 V, giving a peak potential difference between cathodic and anodic peaks of about 90 mV, at slow scan rates of 20 mV s^−1^. The potential at which these redox processes occur varies depending on the chemical environment of the porphyrin. The voltammogram of Figure 3B shows the electrochemical response of TDAP in the presence of TCPP at the same concentration (1 × 10^−5^ M). A reduction peak can be observed at about −0.360 V, presumably due to the presence of a less electrochemically stable product. It is worth noting that the reduction and oxidation potentials reported above (Figure 3A) for the TDAP alone shifted to less negative potentials by about 70 (±5) mV in the presence of TCPP; this may be attributed to the high electron-withdrawing of the TCPP combined with TDAP, providing evidence for the electrostatic interaction between TDAP and TCPP.

**Scheme 3:**
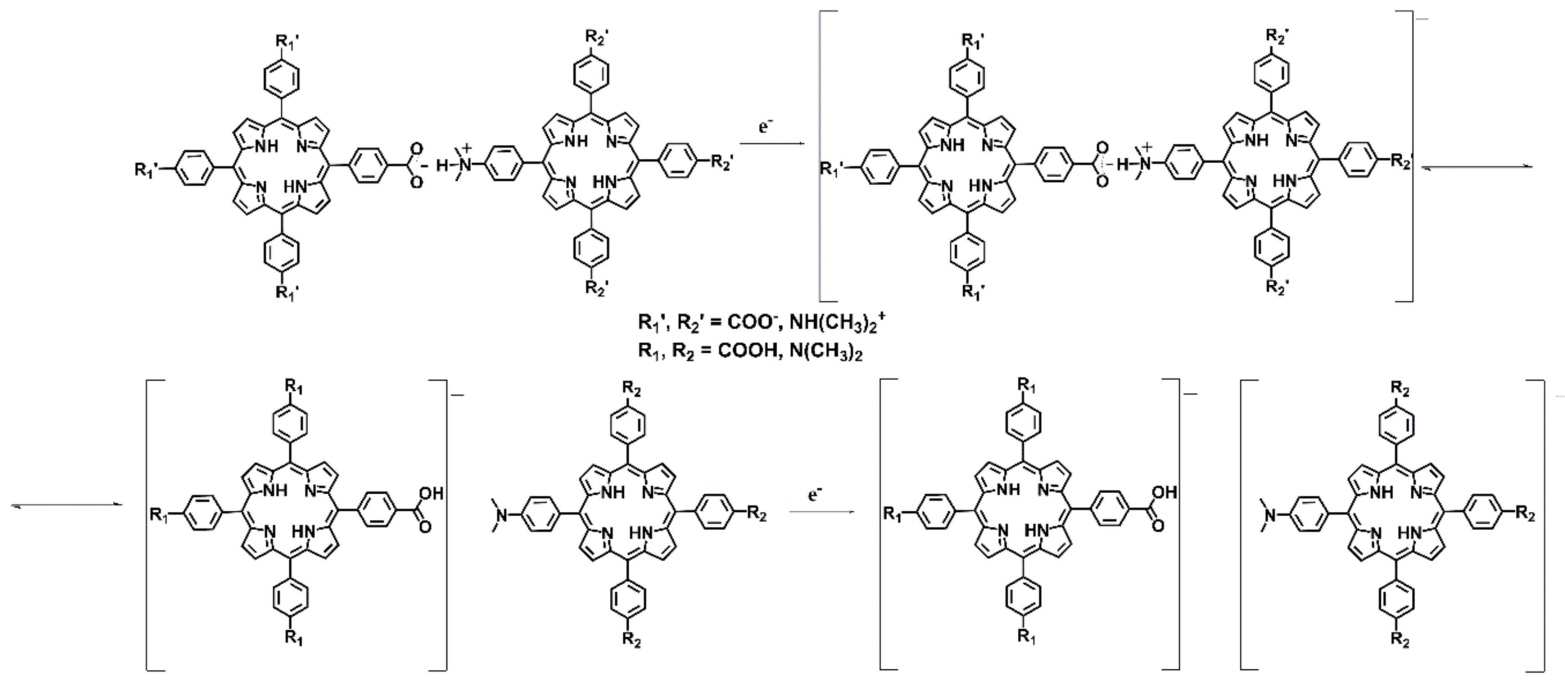
Proposed mechanism of the redox behavior of the supramolecular compound TDAP-TCPP.

**Figure 4:**
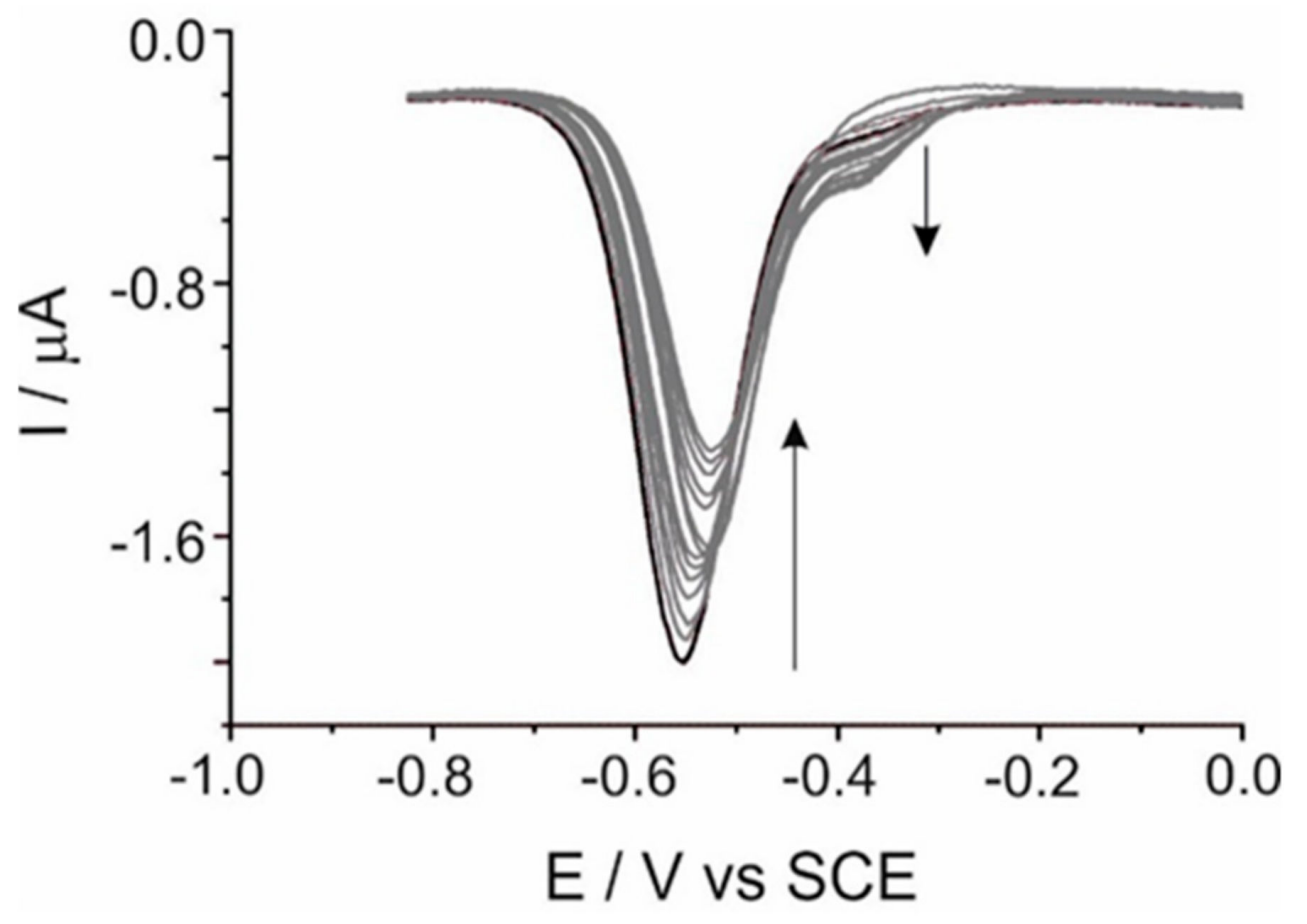
Differential pulse voltammograms of TDAP (solid black line) and the corresponding evolution of the redox signal with increasing amounts of TCPP (solid gray lines). Scan rate of 10 mV s^−1^.

Apparently, the self-assembled product is more easily reduced than the TDAP alone, and this product is not possible to oxidize back. These two facts reflect the stability of the reduced product. Since the reduction at about −0.360 V seems to be irreversible, we hypothesize that the acid-base interactions are broken at this potential. The TDAP redox signal appears at less negative potentials as a consequence of its interaction with the reduced TCPP after the breaking of the assembled compound, making it less stable. The proposed mechanism of the redox behavior for the formation of the molecular assembly is shown in Scheme 3. Additional evidence for this mechanism comes from the monitoring of the TDAP-TCPP titration, explored by differential pulse voltammetry. The voltammetric profiles and response of the system is illustrated in Figure 4. Each individual addition of TCPP is monitored after equilibration. At a scan rate of 20 mV s^−1^, the initial measured redox signal appeared at about −0.55 V with a corresponding cathodic current of −1.8 µA. As an increased amount of TCPP is added, the magnitude current decreases, and the peak potential is shifted to less negative values (about 30 mV). The decreasing of the current is accompanied by the rise of a new redox signal at about −0.370 V, which correlates well with the peak observed by cyclic voltammetry. The decreased current and the emerging redox signal can be associated with the formation of the assembled nanostructure. It is expected that the TDAP current decreases as the quantity of TCPP increases, since some of the TDAP molecules are being shared with the TCPP to form the supramolecular assembly via electrostatic interaction, and the new assembly has a different redox potential, which indicates less stability (compared to TDAP) to the applied potential.

**Figure 5:**
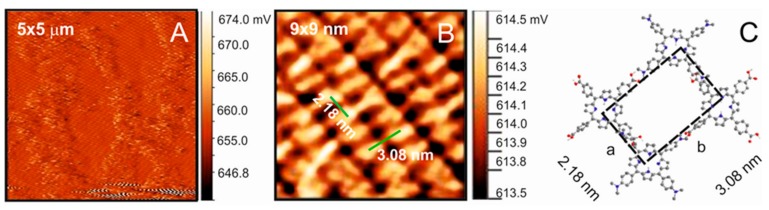
(**A**) Low-resolution AFM image of the assembly on the gold substrate. (**B**) High-resolution AFM image at 9 nm^2^ showing the arrangement of the TDAP-TCPP assembly. On the x-axis, the distance is 2.18, and on the y-axis it is 3.08 nm, measured in the areas marked with green lines. (**C**) Top view of the proposed structural model for the TDAP-TCPP adlayer.

The structural properties of the supramolecular self-assembly were investigated by atomic force microscopy (AFM) in air, in contact mode. Figure 5A shows a typical large-scale AFM image of the self-assembled TDAP-TCPP layer. Gold terraces are completely covered by large molecular domains forming networks, which are separated by areas that form multilayers on the top of substrate defects. High-resolution experiments, such as that in Figure 5B, revealed structural details of the porphyrins forming domains with a long-range ordered structure on Au(111). Due to their large planar π system, it is expected that porphyrins adopt a flat orientation in order to maximize the π bonding to the gold surface. However, a careful inspection of the corresponding cross-section in the high-resolution images shows that the molecules are not lying completely flat on the surface; instead, they are arranged in a bended position, in such a way that it is easy to form a 2D network.

**Scheme A1:**
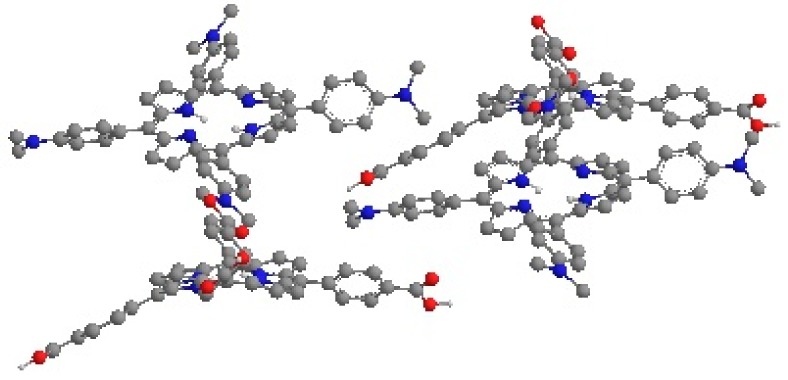
Side view of the structural molecular model proposed in Figure 5C, emphasizing the saddling configuration.

The arrangement of the TDAP-TCPP assembly on the gold substrate shows a nearly rectangular pattern of porphyrins; the dimension of the unit cell as estimated from height profiles gives cell parameters a = 2.18 nm and b = 3.08 nm. The conformation of the porphyrins plays a decisive role in controlling their assembly properties. TCPP tends to adopt a saddling conformation and acute phenyl dihedral angles. The saddling distortion consists of the simultaneous tilting upward of two opposite phenyl rings and the tilting downward of the other two opposite phenyl rings. This configuration in the meso position is affected by the acidity of the peripheral groups. Due to the saddling configuration, two acid groups are anchored to the gold substrate, and the other two are further upward. In order for the first two acid groups to interact with TDAP, they have to slightly force the TDAP amine groups to locate close above them (Scheme A1, Appendix A), which reduces the bonding distance between the neighboring molecules, obtaining a value of 2.18 nm along the x-axis. According to the literature, the average distance between the meso groups of a porphyrin, such as TCPP or TDAP, is around 1.55 nm [14,25]; for the union mode on the y-axis, the sum of the two molecules coincides with the values of parameter b.

Bright porphyrin protrusions define molecular rows, which are aligned in pairs in the short direction of the unit cell (Figure 5B). The fact that such a large structure can be accommodated parallel to the substrate and maintained at standard conditions is due to the fact that the TDAP-TCPP molecule is linked to the gold substrate by weak interactions. These interactions, although weak, are systematically repeated over the entire substrate, keeping the TDAP-TCPP molecule linked without using an external factor as an electrical potential.

Due to their structural flexibility, porphyrins are prone to adopt several geometrical configurations while they are adsorbed on a surface [26]. It seems that the PhCOOH group may adopt a coplanar configuration with the main porphyrin core in order to enable the formation of a hydrogen bond with the dimethyl amino phenyl group of the TDAP. Therefore, a structure is proposed where the relative orientation of TCPP is slightly out of the plane with the carboxylic groups tilted with respect to the surface, as shown by the model in Figure 5C. Most likely, a hydrogen bond via carboxyl groups is playing an important role in this structure, even though the interaction energy is small (vide supra). It has been found that TCPP can form several types of arrays for intermolecular hydrogen bonds that can stabilize molecular networks [14].

Considering that TDAP and TCPP have amino and carboxylate functional groups, respectively, we conclude that the dominant interaction forces for the formation of an ordered structure are non-covalent in nature. The structure is mainly stabilized by electrostatic interactions between the anionic carboxylate group and the protonated amino group of the TDAP.

## 3. Materials and Methods

### 3.1. Synthesis

Meso-tetrakis(4-carboxyphenyl) porphyrin (TCPP) and meso-tetrakis(4-dimethyl amino) porphyrin (TDAP) were synthesized using the Adler–Longo method [27] by mixing pyrrole with 4-formyl benzoic acid and 4-dimethyl amino benzaldehyde, respectively, in propionic acid under reflux conditions. TCPP was purified by chromatography with chloroform and methanol. TDAP was first washed with cold methanol and later purified with chloroform by chromatography.

### 3.2. Spectroscopic Analysis

The changes in the absorption spectra were followed by UV-Vis (Varian, Cary-50, Victoria, Australia) during the spectrophotometric titration of TDAP (ca = 1 × 10^−5^ mol/L) at different (varying) concentrations of TCPP in ethanol, at room temperature, using a quartz cell.

### 3.3. Electrochemical Analysis

To study the molecular interaction in solution by electrochemical analysis, studies were performed with an Epsilon (Bioanalytical Systems) potentiostat-galvanostat. The electrochemical system consisted of a three-electrode glass cell with Ag and Pt wires as a counter and quasi-reference electrode, respectively, although the potentials in figures are represented versus a calomel electrode. Glassy carbon was used as a working electrode. Previously, it was polished using diamond paste (0.1 and 0.04 mµ in diameter, respectively) followed by rinsing with acetone and 0.1 M of NaOH successively. Later, it was rinsed abundantly with Milli-Q water (18 MΩ) several times in an ultrasonic bath. The measurements were performed in tetra butyl ammonium perchlorate (0.1 M TBAP) as a supporting electrolyte in dimethylsulfoxide. All glassware was cleaned in piranha (1:1 H_2_SO_4_:H_2_O_2_, *caution! this mixture is highly reactive!*) followed by copious rinsing with Milli-Q water.

### 3.4. Computational Details

All calculations were performed using Gaussian 09, and the structures were visualized using the Chemcraft 1.6 program. All structures were optimized employing density functional theory (DFT) at the PBE1PBE/cc-pVDZ level of theory with a continuum solvation model based on the charge density of a solute (SDM) model to describe the solvent effect. The ethanol was considered as the solvent. All minima structures were validated by subsequent frequency calculations at the same level of theory. The minimum structures have a set of positive second derivatives. The UV-Vis spectroscopic analysis was carried out using time-dependent density functional theory (TD-DFT) with CAM-B3LYP/cc-pVDZ (SMD, Solvent = Ethanol) from the optimized geometries (PBE1PBE/cc-pVDZ).

### 3.5. AFM Measurements

Structural analysis was carried out by atomic force microscopy (AFM), employing a Nanosurf Naio-AFM microscope provided with silicon carbide tips, and the measurements were performed in contact mode. The Au(111) single crystals used were massive cylinders of 2-mm height and 10-mm diameter, with a nominal miscut angle of 0.1°, from Mateck (Jülich, Germany). Before each experiment, the Au(111) surface was cleaned in piranha and annealed in a butane flame at bright red heat for 10 min. The organic supramolecular adlayer was prepared by immersing the dry Au(111) surface in a 0.1-mM TDAP-TCPP ethanolic solution for 20 s. The modified surface was removed from the assembly solution. Subsequently, it was rinsed thoroughly with ethanol and immediately transferred to the AFM equipment.

### 3.6. Spectroscopic Characteristics of the Synthesized Porphyrins

*Tetra (4-carboxyphenyl) meso porphyrin (TCPP)*: UV-Vis (Et-OH/nm) λ_max_ (S) 415 ε = 136,664, (Q) 520, 550, 595, 645; Fluorescence (Et-OH/nm) λemission 650, 715; IR-FT (KBr/cm^−1^) 3433 t (N–H), 1692 t (C=O acid.); FAB^+^-MS (*m*/*z*) M^+^ 790 (calcd. for TCPP 790.21).

*Tetra (4-dimethyl amino phenyl) meso porphyrin (TDAP)*:UV-Vis (Et-OH/nm). λ_max_ (S) 435 ε = 21642, (Q) 530, 575, 660; Fluorescence (Et-OH/nm) λemission 679; IR-FT(KBr/cm^−1^) 3406 t (N–H), 2974 t (C–H), 1558 t (C=N); FAB-MS (*m*/*z*) M^+^ 787 (calcd. for TDAP 787.01).

## 4. Conclusions

The presented studies demonstrated the formation of supramolecular self-assembled ordered arrays of TDAP-TCPP by simple combination in solution of the building blocks. Apparently non-covalent interactions (hydrogen bonds) are the main driving force for the formation of 2D structures. The binding ability of TDAP and TCPP provides the opportunity to create self-assembled ordered arrays on gold surfaces; this triggers the possibility to explore other metallic or semiconducting substrates to assemble supramolecular arrays based on weak interactions.

## Appendix A

### Computational Results

The UV-Vis spectra performed using time-dependent density functional theory (TD-DFT) are shown in Figure A1. Once TDAP was in close contact with four molecules of protonated benzoic acid (to simulate a TCPP molecule), a shift towards higher values was observed. The corresponding energy values appear in Table A1.

**Table A1:** Frontier Molecular Orbitals, values of the energy in eV and GAP (HOMO-LUMO).

OM	TDAP	BA	BAA	BAA*	BAAA	BAAAA
HOMO-1	−6.3779	−6.3893	−6.4069	−6.4132	−6.4313	−6.4484
HOMO	−5.7961	−5.7808	−5.8785	−5.8594	−5.9967	−6.0590
LUMO	−1.5840	−1.6035	−1.6237	−1.6314	−1.6491	−1.6700
LUMO+1	−1.5600	−1.5781	−1.6035	−1.5933	−1.6264	−1.6518
GAP(H-L)	4.2122	4.1773	4.2547	4.2279	4.3476	4.3891
